# COVID-19 and income inequality in OECD countries

**DOI:** 10.1007/s10198-021-01266-4

**Published:** 2021-02-15

**Authors:** John Wildman

**Affiliations:** Population Health Sciences Institute, Newcastle University, Newcastle upon Tyne, NE1 7RU UK

**Keywords:** COVID-19, Income inequality, OECD, Regression, I10, I14

## Abstract

**Objective:**

To determine the association between income inequality and COVID-19 cases and deaths per million in OECD countries.

**Methods:**

Cross-sectional regression methods are used to model the relationship between income inequality, as measured by the Gini coefficient, and COVID-19 reported cases and deaths per-million.

**Results:**

The results demonstrate a significant positive association between income inequality and COVID-19 cases and death per million in all estimated models. A 1% increase in the Gini coefficient is associated with an approximately 4% increase in cases per-million and an approximately 5% increase in deaths per-million.

**Conclusions:**

The results demonstrate that countries with high levels of income inequality have performed significantly worse when dealing with the COVID-19 outbreak in terms cases and deaths. Income inequality is a proxy for many elements of socioeconomic disadvantage that may contribute to the spread of, and deaths from, COVID-19. These include poor housing, smoking, obesity and pollution.

**Policy Implications:**

The findings suggest the importance of closing the gap in income inequality and improving the health and incomes of the poorest and most vulnerable groups.

## Introduction

As OECD countries lift their lockdowns, emerging from the first wave of the COVID-19 pandemic, it is an excellent time to consider factors that may have affected COVID-19 outcomes before the advent of any secondary spikes. Why have some OECD countries performed much better than others?

According to the European Centre for Disease Prevention and Control (ECDC), up to the 9th June 2020 Germany had 184,543 cases and 8711 deaths compared to the UK’s 287,399 cases and 40,597 deaths [1]. How does the UK, with a much smaller population, have nearly 60% more cases and five times as many recorded deaths as Germany?

Such heterogeneity between countries suggest that when it comes to catching or dying from COVID-19 we are not ‘all in it together' (e.g. Guterres 2020 [2]). However, while everyone may be affected in some ways, there are always those who are more vulnerable than others.

This paper is the first to investigate whether income inequality has played a role in explaining the differences in COVID-19 outcomes across countries. Although the immediate response to the pandemic has, quite correctly, focused on the biological and medical factors relating to COVID-19, it is important to remember that disease outcomes are related to socioeconomics factors. Already the role of inequality has been considered within individual countries regarding the impact of COVID-19, with van Dorn et al. (2020) noting that it is a pandemic falling on the most vulnerable people in the US [3].

A key starting point is the relative income hypothesis (RIH): in developed countries, income inequality is more important in determining health outcomes than absolute income [4–8]. The RIH provides the initial motivation behind this analysis, examining the relationship between income inequality and health outcomes, but this paper is not simply attempting to revisit the RIH.

This paper is interested in the relationship between income inequality and COVID-19 on the basis of two key arguments:

First, the importance of income, be it relative or absolute. The RIH could be the correct mechanism. There is considerable research linking income inequality to a whole range of outcomes and it is possible that income inequality has played a direct role in COVID-19 outcomes [9]. Furthermore, even if the RIH is not the correct mechanism, and absolute income is the key driver behind health outcomes, if any significant relationship between income inequality and COVID-19 is found it demonstrates that the distribution of income matters [6].

Secondly, Income inequality is a proxy for many elements of socioeconomic disadvantage [8], many of which may contribute to the spread of, and deaths from, COVID-19. These include poor housing quality in deprived and polluted urban areas that is often cramped and damp, the latter being associated with lung disease [10]; lifestyle factors, such as smoking, obesity, nutrition and exercise, all of which are associated with the identified COVID-19 risk factors; education, which is associated with health outcomes and compliance with public health messages [11]. These factors all suggest that some countries have populations that are less deprived and so better placed at coping with COVID-19.

## Methods

This paper uses data on COVID-19 from the ECDC [2]. This data contains recorded daily cases and deaths across the globe along with population counts. Recorded cases and deaths are summed up to 18 May 2020 (for Spain data are only available for the 17 May and that date is used) to give overall number of cases and deaths for each country. These are converted to cases and deaths per million of population. I link this data with World Bank data on income inequality, as measured by the Gini coefficient, and GDP per capita [12]. The Gini coefficient runs from zero to one hundred, with zero being complete equality and one hundred being complete inequality. This is a widely used measure in the analysis of the RIH. New Zealand’s Gini coefficient is taken from the New Zealand Government statistics [13].

Extra data from the OECD is collected on the age, proportion of the population aged over 65, and health status, average life-expectancy at birth, of populations. And the data on lockdowns and responses were taken from the Oxford COVID-19 Government Response Tracker [14].

Although COVID-19 data are current, GDP per capita data are taken from 2018, the most complete year for our sample of countries. For income inequality, we use the value from the nearest year, going back as far as 2012. Using this approach, 80% of countries have data for income inequality from between 2016 and 2018.

The focus on OECD countries ensures that the data comes from economically developed, wealthy countries, with good education and health systems, where we can be confident of good recording practices. OECD countries are broadly comparable and provide some sense of homogeneity.

COVID-19 cases and deaths are regressed on GDP per capita, to control for overall country wealth, and the Gini coefficient. The two basic regressions are:

1 $${\mathrm{Covid}\_\mathrm{outcome}}_{i}= {\alpha }_{0}+ {\alpha }_{1}\mathrm{lngdp}+ {\alpha }_{2}\mathrm{NE}+{\alpha }_{3}{\mathrm{case}\_\mathrm{days}}_{i}+ {\alpha }_{4}{\mathrm{gini}}_{i}+ {u}_{i}$$

2 $${\mathrm{ln }(\mathrm{Covid}\_\mathrm{outcome}}_{i})= {\alpha }_{0}+ {\alpha }_{1}\mathrm{lngdp}+ {\alpha }_{2}\mathrm{NE}+ {\alpha }_{3}{\mathrm{case}\_\mathrm{days}}_{i}+ {\alpha }_{4}{\mathrm{lngini}}_{i}+ {u}_{i}$$

*Covid_outcome* represents either cases per-million or deaths per-million for country *i* (or its natural logarithm), *lngdp* is natural logarithm of GDP per capita, *NE* is a dummy variable which equals one if the country is not in continental Europe and zero otherwise, *case_days* is the number of days since the first recorded case in each country, according to the ECDC, and *gini* (*lngini*) i*s* the (natural logarithm of the) Gini coefficient. All models were estimated using robust standard errors.

**Fig. 1:**
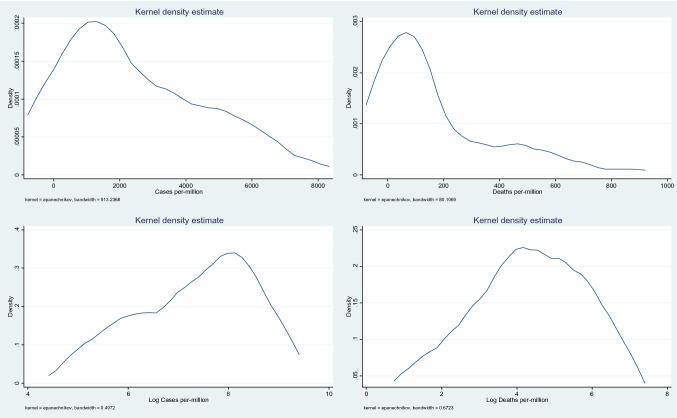
Density plots

Figure 1 shows the distribution of the dependent variables. The untransformed data has a long right hand tail. Following the log transformation, the distributions, especially for the log of deaths, are much closer to normal. As an extra robustness check, models for the untransformed dependent variables were estimated using Poisson regression and the results for income inequality were qualitatively similar. Furthermore, with a small sample, it is possible that we have influential observations. In order to investigate whether this is the case a Jackknife procedure was estimated and again, the results were largely unchanged.

## Results

The summary statistics are given in Table 1 and the regression results are presented in Table 2 (Cases per-million) and Table 3 (Deaths per-million). All of the models are highly significant overall, with the *R*^*2*^ ranging from 0.419 to 0.617, meaning that our models explain between 41.9% and 61.7% of the variation in cases and deaths per-million. The results demonstrate a generally positive (and significant for Cases) association between GDP per capita and the number of cases and number of deaths per-million. Even though these are all OECD countries, the results may demonstrate that countries with more resources suffer more cases because of their greater exposure to international travel and trade, or that they have better recording and reporting systems. The association with deaths is weaker and less significant, highlighting that while high resource levels may be associated with more cases, they are largely unrelated to the number of deaths. Countries not in the continent of Europe have, on average, lower cases and deaths.

**Table 1:** Summary statistics

	Mean	sd	Min	Max
Cases per-million	2474.21	2077.79	136.02	7413.36
Deaths per-million	175.03	214.06	4.08	841.27
GDP per capita	41,889.82	24,240.02	9370.18	116,639.89
Not in Europe	0.28	0.45	0.00	1.00
Days since first case reported	112.31	18.56	88.00	148.00
Proportion of population aged over 65	17.75	4.01	7.22	27.58
Life Expectancy at birth	80.72	2.56	74.80	84.20
Days from first case to lockdown	31.11	25.42	2.00	115.00
Maximum lockdown stringency index	78.80	11.89	46.00	96.00
Gini	32.70	5.22	24.20	45.40

**Table 2:** Cases Regression results

	(1)	(2)	(3)	(4)	(5)	(6)	(7)	(8)
	Cases per mill	Cases per mill	Cases per mill	Cases per mill	Ln Cases per mill	Ln Cases per mill	Ln Cases per mill	Ln Cases per mill
Log GDP	1925.2***	1666.3***	1049.0	866.0	0.914***	0.737***	0.441	0.355
	(377.8)	(353.7)	(710.7)	(813.5)	(0.219)	(0.213)	(0.393)	(0.384)
Not in Europe	− 1903.9**	− 2844.5***	− 3155.8***	− 3179.7***	− 1.268**	− 1.997***	− 2.143***	− 1.799***
	(727.4)	(650.6)	(644.9)	(922.6)	(0.518)	(0.376)	(0.410)	(0.535)
Case Days	0.795	25.90	20.14	22.45	− 0.00188	0.0168	0.0140	0.0301*
	(15.00)	(18.63)	(20.78)	(27.29)	(0.00906)	(0.0107)	(0.0118)	(0.0158)
Age		− 203.9**	− 225.9***	− 227.5**		− 0.149***	− 0.160***	− 0.139**
		(84.63)	(77.71)	(87.56)		(0.0496)	(0.0474)	(0.0517)
Life Expectancy			243.1	277.8			0.116	0.143
			(198.1)	(243.0)			(0.102)	(0.111)
Days to lockdown				− 11.36				− 0.0238
				(27.07)				(0.0145)
Max lockdown				− 43.02				− 0.0290**
				(29.25)				(0.0128)
Gini	282.8***	246.1***	259.7***	272.5***				
	**(70.83)**	**(73.56)**	**(73.42)**	**(77.29)**				
Log Gini					4.532***	3.814***	4.007***	3.879***
					**(1.204)**	**(1.293)**	**(1.275)**	**(1.352)**
Constant	− 26,491.1***	− 21,517.2***	− 34,002.5***	− 31,772.0**	− 17.42***	− 12.33**	− 18.70**	− 18.79**
	(3504.8)	(3823.3)	(9695.8)	(11,589.7)	(3.888)	(5.246)	(7.200)	(7.415)
*N*	36	36	36	35	36	36	36	35
*R*^2^	0.457	0.526	0.562	0.588	0.419	0.547	0.575	0.617
F	21.05	23.61	20.07	15.05	12.80	15.53	15.96	13.95

Bold values indicates the results for the Gini (lngini)

Standard errors in parentheses

*p < 0.10, **p < 0.05, ***p < 0.01

**Table 3:** Deaths Regression results

	(1)	(2)	(3)	(4)	(5)	(6)	(7)	(8)
	Deaths per mill	Deaths per mill	Deaths per mill	Deaths per mill	Ln Deaths per mill	Ln Deaths per mill	Ln Deaths per mill	Ln Deaths per mill
Log GDP	36.07	9.077	− 43.96	− 19.40	0.531*	0.354	0.0469	0.162
	(32.36)	(37.76)	(48.96)	(54.21)	(0.281)	(0.292)	(0.479)	(0.466)
Not in Europe	− 299.2***	− 397.2***	− 424.0***	− 446.4***	− 2.406***	− 3.139***	− 3.290***	− 3.114^***^
	(79.23)	(98.45)	(94.17)	(95.54)	(0.651)	(0.503)	(0.535)	(0.866)
Case Days	6.299***	8.915***	8.421***	7.037*	0.0232**	0.0420***	0.0392**	0.0441*
	(1.858)	(2.725)	(2.782)	(3.464)	(0.0109)	(0.0150)	(0.0158)	(0.0247)
Age		− 21.25**	− 23.14**	− 22.77**		− 0.150**	− 0.161**	− 0.127
		(9.369)	(9.412)	(10.77)		(0.0647)	(0.0661)	(0.0790)
Life Expectancy			20.89*	12.61			0.120	0.0605
			(10.61)	(13.32)			(0.127)	(0.138)
Days to lockdown				2.308				− 0.00822
				(2.637)				(0.0231)
Max lockdown				4.377				− 0.00462
				(2.735)				(0.0217)
Gini	18.75***	14.92**	16.09**	16.53**				
	**(5.871)**	**(6.788)**	**(6.701)**	**(7.669)**				
Log Gini					5.986***	5.263***	5.464***	5.673**
					**(1.662)**	**(1.899)**	**(1.818)**	**(2.111)**
Constant	− 1440.0***	− 921.5**	− 1994.2***	− 1856.5**	− 24.03***	− 18.90**	− 25.49**	− 23.19**
	(313.8)	(415.9)	(627.5)	(710.2)	(5.886)	(8.037)	(9.344)	(9.587)
*N*	36	36	36	35	36	36	36	35
*R*^2^	0.439	0.509	0.535	0.556	0.430	0.500	0.516	0.531
*F*	10.26	8.714	11.96	9.160	9.668	14.96	14.23	12.25

Bold values indicates the results for the Gini (lngini)

Standard errors in parentheses

*p < 0.10, **p < 0.05, ***p < 0.01

The number of days since the first reported case is either positive, or very close to zero. This variable helps to control for the stage of the virus. It is unsurprising that more cases and deaths are generally associated with more days since the first reported case.

It is notable that the estimated coefficients attached to the *Gini* coefficient are all positive and significant (all but one at the 5% level at least). These results suggest a clear association between income inequality and COVID-19 cases and deaths. In order to ease interpretation columns (5)–(8) in both tables present log–log models, these allow the estimated coefficients to be interpreted as elasticities. For example, column (5) of Table 3 shows that a 1% increase in income inequality is associated with a 5.986% increase in deaths per-million. To put these numbers into context, a one standard deviation increase in the *Gini* coefficient equates to an approximately 16% increase of the mean value of the *Gini* (32.70–37.99). Using the results from column (5), this 16% increase in income inequality is associated with an approximately 96% increase in the deaths per-million—an extra 343 deaths per-million at the mean.

It is possible that countries with higher income inequality have older populations, leading to more deaths, although not necessarily more cases. To investigate whether this is the case we included the proportion of the population aged over 65 as an extra regressor, see columns (2) and (6) in Tables 2 and 3. The estimates for the Gini coefficient, while slightly lower in magnitude, remain positive, large and significant. This suggests that population age is not a confounder for the effect of income inequality.

It may be the case that the underlying average health of the population is a confounding factor. The average life expectancy from 2017 (2016 in the case of Chile) is included as an extra regressor in the model. There is no clear a priori hypothesis as to the direction of the sign of this variable, it could be positive or negative. Higher life-expectancy is a sign that there are more elderly individuals who may be more susceptible to COVID-19. However, high life-expectancy is related to a healthy population with lower probabilities of death at younger age groups, as compared to low life-expectancy countries. In this case there may be a more robust population. The problem is that we do not observe the distribution of health. What the results demonstrate is that after including life-expectancy the estimated coefficients attached to the Ginis are still positive, large and significant.

Finally, I consider the possibility that countries with higher income inequality implemented earlier and/or more stringent lockdowns. The Government Response data provide a stringency index for the degree of lockdown [14]. The stringency index is an average of individual component indicators that relate to the type and severity of lockdown, including school closures, stay at home advice, travel advice etc. Two extra variables are included as regressors, the maximum value of this index for each country, measuring how stringent the lockdown was at the strictest point, and the number of days between the first confirmed case and the implementation of a lockdown at the level of Sweden, based on the value of the stringency index. Sweden was chosen since it was quite a light touch lockdown, meaning that the number of days until lockdown is a conservative estimate for most countries. There was no data available for Latvia. The results (columns (4) and (8)) show that the estimates for income inequality are still large, positive and significant.

## Discussion

### What may be driving the association between income inequality and COVID-19 outcomes?

The results demonstrate a strong association between income inequality and the number of COVID-19 deaths. We can consider a number of reasons why this may be the case. First, we suggest two unlikely reasons for the association, before moving to more credible explanations.

COVID-19 cases and deaths are higher in countries with high income inequality because countries with high income inequality are at a later stage of the virus as compared to more equal countries. There is no reason to believe that this is the case and there are no compelling reasons as to why high income inequality should be associated with an earlier onset of COVID-19 once GDP has been controlled for. Also, the results from our models, when including days since the first case seem to rule out this reason. We also demonstrate that the link is not caused by time since lockdown, nor by the stringency of the lockdown.

COVID-19 cases and deaths are higher in countries with high income inequality because more unequal countries have better records than more equal countries. Again, after controlling for GDP, there is no reason to believe that more unequal countries are better at recording cases and deaths than more equal countries. As countries are emerging from lockdown it is clear that governments believe that the first wave of the virus has passed, so it would be reasonable to expect that records on deaths and cases are accurate across OECD countries.

More credibly, the positive association between income inequality and COVID-19 outcomes may be evidence of the relative income hypothesis. Wider income inequalities lead to worse health outcomes. Although aggregate data may not be the most appropriate way of providing evidence in favour of the RIH, our results do not preclude this from being a possible explanation.

Alternatively, income inequality could, as hypothesised by Wilkinson (1996) [15] be a proxy for social capital [16] and the investment in, and popular support of, public services. Countries with a more even distribution of income may have a stronger sense of the public sector, with well-funded safety-nets and services. One of the reasons suggested for Germany’s success in large scale testing and limiting deaths was both its network of laboratories for testing and its strong federal public health network [17]. This may suggest that countries with low levels of income inequality were simply more prepared and were in a stronger position to cope with the COVID-19 crisis. Perhaps, it is also the case that stronger social capital also leads to individuals following lockdown restrictions more stringently.

Finally, income inequality is, as suggested above, linked to poverty and socioeconomic disadvantage. Income inequality acts as a marker for the proportion of the population with low absolute incomes, which would be hidden by GDP per capita. Countries with high income inequality have a large proportion of their population living precarious lives, with low income and insecure jobs. Those at the bottom end of the income distribution are vulnerable, have many comorbidities and live in poor quality houses in highly polluted areas, and there is a clear association between poor health, as measured by mortality, and deprivation [18, 19]. Furthermore, the link between inequality and poor health has been widely considered in the UK [20], and the current COVID-19 pandemic has disproportionately affected many of the most deprived areas of England [21]. Overall, it is perhaps most likely that a combination of social capital, poverty and poor public health contribute to the number of COVID-19 cases and deaths.

### Public health implications

Very little is known about the association between income inequality and COVID-19. There has been increasingly robust data demonstrating that individuals from low socioeconomic backgrounds are more vulnerable to COVID-19 [22] and that some of the major risk factors, especially obesity, are much more prevalent in those individuals from disadvantaged backgrounds [23]. It is also becoming clear that, for deaths per-million, the poorest regions are suffering the most. In the UK the four areas with the highest death rates [24] are all located in the North East of England, one of the poorest areas of the country and, given the later onset of the disease (the UK had its first confirmed death registered on the 2 March, while for Gateshead, in the North East of England, the first deaths were not registered until the second week of April), one that would have potentially benefited more from the lockdown.

The paper has identified income inequality as a factor that needs to be addressed as part of preparing for future pandemics. The public health implications are stark; more equal countries seem to have coped with the COVID-19 crisis better. Inequalities make populations vulnerable. Countries with wider inequalities have more vulnerable populations, with weaker public services, and living and working conditions that put them most at risk from illness, disease and mortality [15], including COVID-19.

### Limitations of this study

This study takes place during the initial phase of COVID-19 among OECD countries, although most countries are now starting to emerge from their lockdowns and daily death rates are (at the time of writing), in the main, falling. At this stage, there are a number of threats to the validity of this study, e.g. the data recording is not comparable, the outbreaks occurred at different times and the lockdowns were not comparable. This paper has tried to accommodate the latter two elements, and finds no compelling evidence that undermines the key results. In order for differences in recording methods to invalidate the results they would have to be correlated with income inequality. There is no reason why more accurate recording, or a willingness to attribute cases and deaths to COVID-19 is systematically associated with income inequality. If anything recording is most likely to be correlated with GDP, which is controlled for. If recoding is randomly associated with income inequality, as is more likely, then the results from an OLS regression will still be unbiased.

The use of aggregate data always raises challenges when analysing relationships that are defined at the individual level. There are a number of problems to be considered. Firstly, the results are not causal, it is not possible to demonstrate that income inequality causes the COVID-19 outcomes—this would be a demonstration of the relative income hypothesis. Although the levels of income inequality pre-date the outcomes there are confounding factors that may be correlated with both income inequality and COVID-19 outcomes. In fact, this is very much the point of this paper, that the factors correlated with income inequality are important for COVID-19 outcomes. And that addressing income inequality will, either directly or indirectly, improve health outcomes.

Secondly, the use of aggregate data is always problematic. Relationships between risk of death and key characteristics are often nonlinear and aggregating to country level analysis raises potential problems of functional form and omitted variable bias. Furthermore, variables are measured as averages, whereas distributions would often be of interest. For example, the level of health inequality in each country would be an interesting extra variable but no such unified regularly measured variable exists. However, it is only by considering aggregate level data that it is possible to compare the performances of different countries. It is also the case that income inequality is by definition an aggregate measure.

## Conclusion

As countries emerge from their lockdowns the results from this paper suggest that we are not ‘all in it together’. Just as the economic consequences are likely to hit the most vulnerable, so are the health consequences [21, 25]. It may be the case, as the world re-evaluates in the aftermath of COVID-19, that in future, a goal of government should be to reduce inequalities and improve the underlying health of their populations.

## Data Availability

Links to the data sources are provided—all data is free to access.

## Code availability

Modelling code is available on request.
